# Supplementary material for: Estimating density of native carnivores in central Chile landscapes using a simulated movement model, cameratrapR: insights on their potential exotic prey dietary subsidy
Source: PeerJ. 2025 Sep 1;13:e19946. doi: 10.7717/peerj.19946 (PMC12422279; doi:10.7717/peerj.19946)
Supplement: Supplemental Information 1 [file peerj-13-19946-s001.docx]

| Species* | Mean footprint chain length (km) | Home range  ± SD (km^2^) | Segment length  ± SD (m) | SD of Angular deflection | Mean No. of  steps per chain | No. of chains |
| --- | --- | --- | --- | --- | --- | --- |
| Cape hare | 0.7 | 0.25±0.11 | 13.49±13.27 | 37.09 | 1557 | 3 |
| Red deer | 4.9 | 1.95±1.15 | 15.46±10.21 | 40.43 | 9508 | 6 |
| Roe deer | 2.7 | 0.66±0.69 | 13.82±6.77 | 43.08 | 5861 | 25 |
| Moose | 4.8 | 4** | 55.97±43.58 | 38.27 | 2572 | 7 |
| Chinese goral | 3.0 | 9.2±0.62 | 17.20±34.78 | 32.94 | 1570 | 3 |
| Wild boar | 3.6 | 1.15±1.28 | 16.13±11.01 | 43.21 | 6696 | 15 |
| Red fox | 6.2 | 2.2 | 14.50±19.02 | 21.89 | 3724 | 1 |
| Corsac fox | 25.7 | 0.9 | 12.92±27.48 | 26.75 | 59652 | 1 |
| Raccoon dog | 1.3 | 2** | 12.12±5.43 | 30.28 | 3218 | 3 |
| Eurasian lynx | 2.3 | 8.81±13.21 | 21.08±13.40 | 50.15 | 3273 | 6 |
| Leopard cat | 1.3 | 4** | 16.97±12.73 | 31.93 | 2298 | 4 |
| Yellow-throated marten | 2.6 | 2** | 13.98±7.02 | 52.27 | 5579 | 4 |
| Sable | 2.4 | 2** | 11.27±6.91 | 53.20 | 6389 | 17 |
| Siberian weasel | 1.0 | 1.2±0.04 | 13.06±31.54 | 50.89 | 2223 | 2 |

* Latin names for the species: cape hare (*Lepus capensis*), red deer (*Cervus elaphus*), roe deer (*Capreolus capreolus*), moose (*Alces alces*), Chinese goral (*Naemorhedus griseus*), wild boar (*Sus scrofa*), red fox (*Vulpes vulpes*), corsac fox (*Vulpes corsac*), raccoon dog (*Nyctereutes procyonoides*), Eurasian lynx (*Lynx lynx*), leopard cat (*Prionailurus bengalensis*), Yellow-throated marten (*Martes flavigula*), sable (*Martes zibellina*), Siberian weasel *(Mustela sibirica*).

** The areas were artificially adjusted based on expert knowledge.
