# Supplementary figures and images for: Estimating density of native carnivores in central Chile landscapes using a simulated movement model, cameratrapR: insights on their potential exotic prey dietary subsidy
Source: PeerJ. 2025 Sep 1;13:e19946. doi: 10.7717/peerj.19946 (PMC12422279; doi:10.7717/peerj.19946)

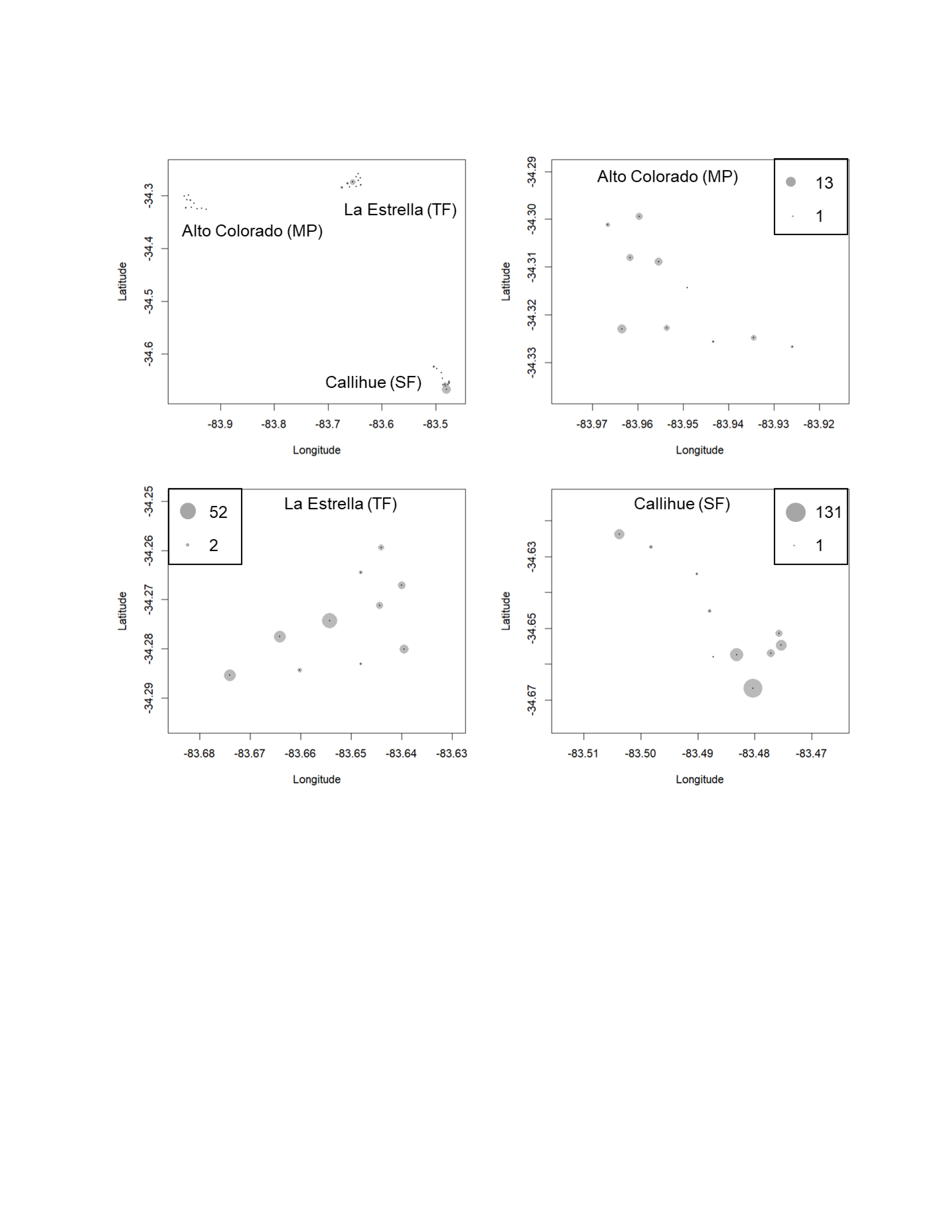

Supplement: Supplemental Information 2 [file peerj-13-19946-s002.jpg]

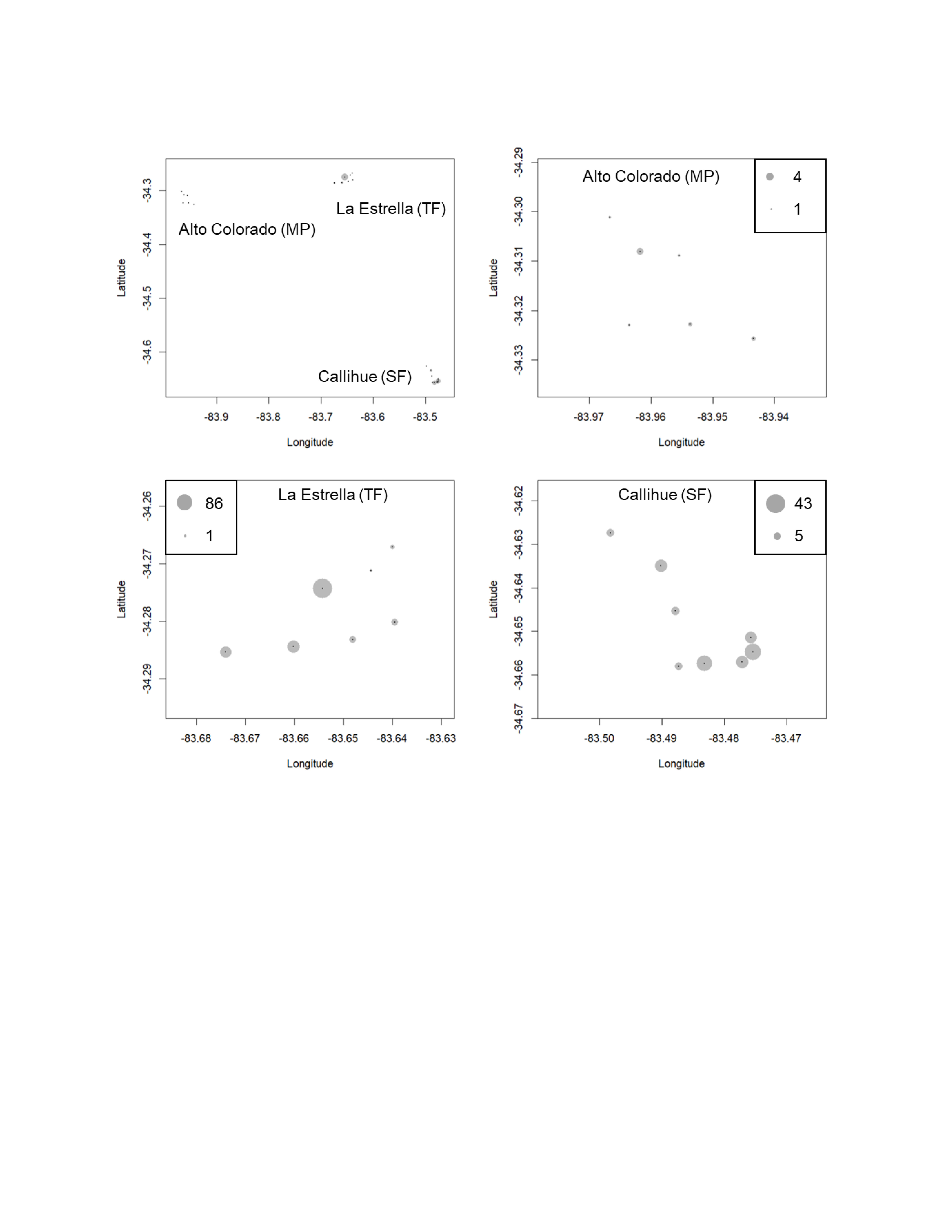

Supplement: Supplemental Information 3 [file peerj-13-19946-s003.jpg]

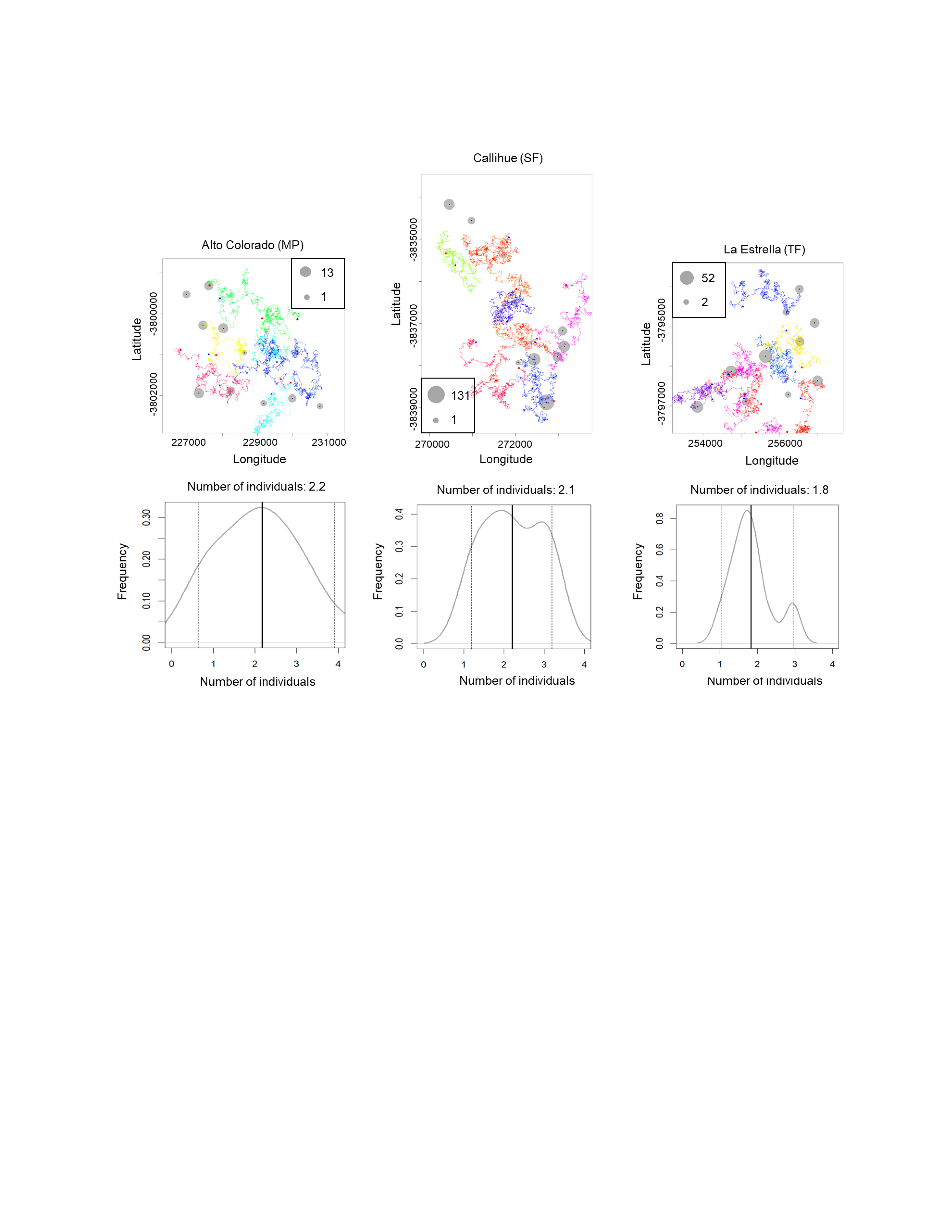

Supplement: Supplemental Information 4 [file peerj-13-19946-s004.jpg]

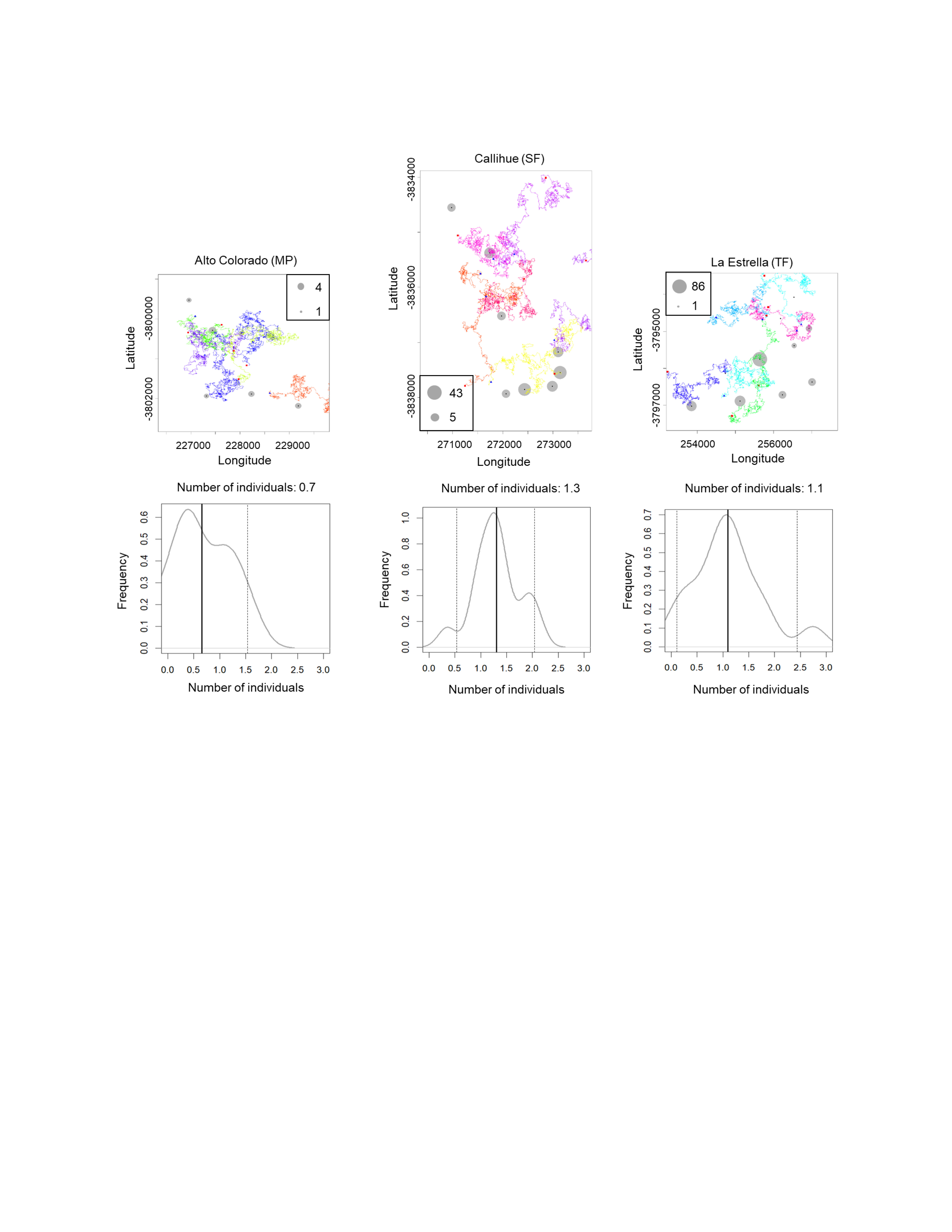

Supplement: Supplemental Information 5 [file peerj-13-19946-s005.jpg]
